# Assessment of Carotid Stiffness and Strain Parameters Using Speckle Tracking Strain Ultrasonography in Rheumatoid Arthritis Patients

**DOI:** 10.31083/RCM27092

**Published:** 2025-04-25

**Authors:** Volkan Tasci, Ali Fuat Tekin, Huseyin Baygin, Alparslan Unsal, Mustafa Gok

**Affiliations:** ^1^Department of Radiology, Silvan State Hospital, 21640 Diyarbakir, Turkiye; ^2^Department of Radiology, Basaksehir Cam Sakura City Hospital, 34480 Istanbul, Turkiye; ^3^Department of Biomedical Engineering, Bogazici University, 34470 Istanbul, Turkiye; ^4^Department of Rheumatology, Merkezefendi State Hospital, 45120 Manisa, Turkiye; ^5^Department of Radiology, Faculty of Medicine, Adnan Menderes University, 09010 Aydin, Turkiye; ^6^Faculty of Medicine and Health, University of Sydney, Sydney Medical School and School of Health Sciences, Sydney, NSW 2050, Australia

**Keywords:** arterial wall stiffness, arterial wall strain, cardiovascular disease, carotid intima-media thickness, rheumatoid arthritis, speckle tracking carotid strain, ultrasonography

## Abstract

**Background:**

Rheumatoid arthritis (RA) is a chronic, systemic autoimmune disease characterized by progressive joint deformity and increased mortality. RA patients typically exhibit elevated plasma levels of inflammatory markers, contributing to endothelial dysfunction and increased arterial wall stiffness—a recognized marker of subclinical atherosclerosis and heightened cardiovascular risk. This study aimed to evaluate carotid arterial wall stiffness in RA patients using ultrasound (US) imaging modality with speckle tracking carotid strain (STCS) software, a non-invasive method for assessing subclinical cardiovascular disease indicators.

**Methods:**

This analytical case–control study was conducted at Aydin Adnan Menderes University Hospital Department of Radiology and Department of Rheumatology. Patients who met the inclusion criteria were enrolled in the study. Data collection tools included an 11-item case report form developed by the researcher based on relevant literature and carotid US examinations performed.

**Results:**

The study included 143 participants: 75 RA patients (60 female and 15 male) and 68 control subjects (54 female and 14 male). The mean age was 50.9 ± 11.4 years (range: 25.0–74.0) for the RA group and 53.1 ± 12.6 years (range: 20.0–77.0) for the control group. Systolic blood pressure (SBP) and C-reactive protein (CRP) levels (mean ± SD) were 7.4 ± 11.5 in the RA group and 8.6 ± 22.2 in the control group. However, due to a few outliers in the control group, the median CRP was 3.3 mg/L (range: 2.0–71.9) in the RA group versus 2.0 mg/L (range: 0.8–145.0) in the controls. This nonparametric comparison showed significantly higher typical CRP levels in the RA group (*p* < 0.05). All stiffness and strain parameters in axial and longitudinal planes showed statistically significant differences between the two groups (*p* < 0.05), except the circumferential strain parameter “displacement (DP)” (*p* = 0.074). Although no significant correlation was found between the disease activity score (DAS) and any strain or stiffness parameter, the carotid intima-media thickness (CIMT) exhibited a significant positive correlation with disease duration (*p *= 0.001). After adjusting for confounding factors (age, gender, body mass index (BMI), and smoking status) using multivariate linear regression analysis, RA remained a significant predictor for all stiffness and strain parameters, except for the circumferential strain parameter DP.

**Conclusion:**

Applying functional parameters to assess arterial wall stiffness and tension levels provides valuable insights for early detection of cardiovascular disease risk, preceding classical US findings such as increased intima-media thickness (IMT) and plaque formation. While preliminary, our findings from STCS measurements in RA patients show promise in evaluating cardiovascular disease risk in this population and potentially improving long-term outcomes through timely interventions.

## 1. Introduction

Rheumatoid arthritis (RA) is a chronic, systemic inflammatory autoimmune disorder primarily characterized by joint inflammation and progressive joint deformity [1]. In addition to its impact on the joints, RA significantly increases the risk of cardiovascular diseases (CVDs), which are the leading cause of mortality in patients with RA [2]. The cardiovascular mortality rate in RA patients is approximately three times higher than in the general population; this heightened risk is largely attributed to chronic inflammation, which plays a pivotal role in the pathogenesis of both RA and atherosclerosis [3].

**Fig. 1.**
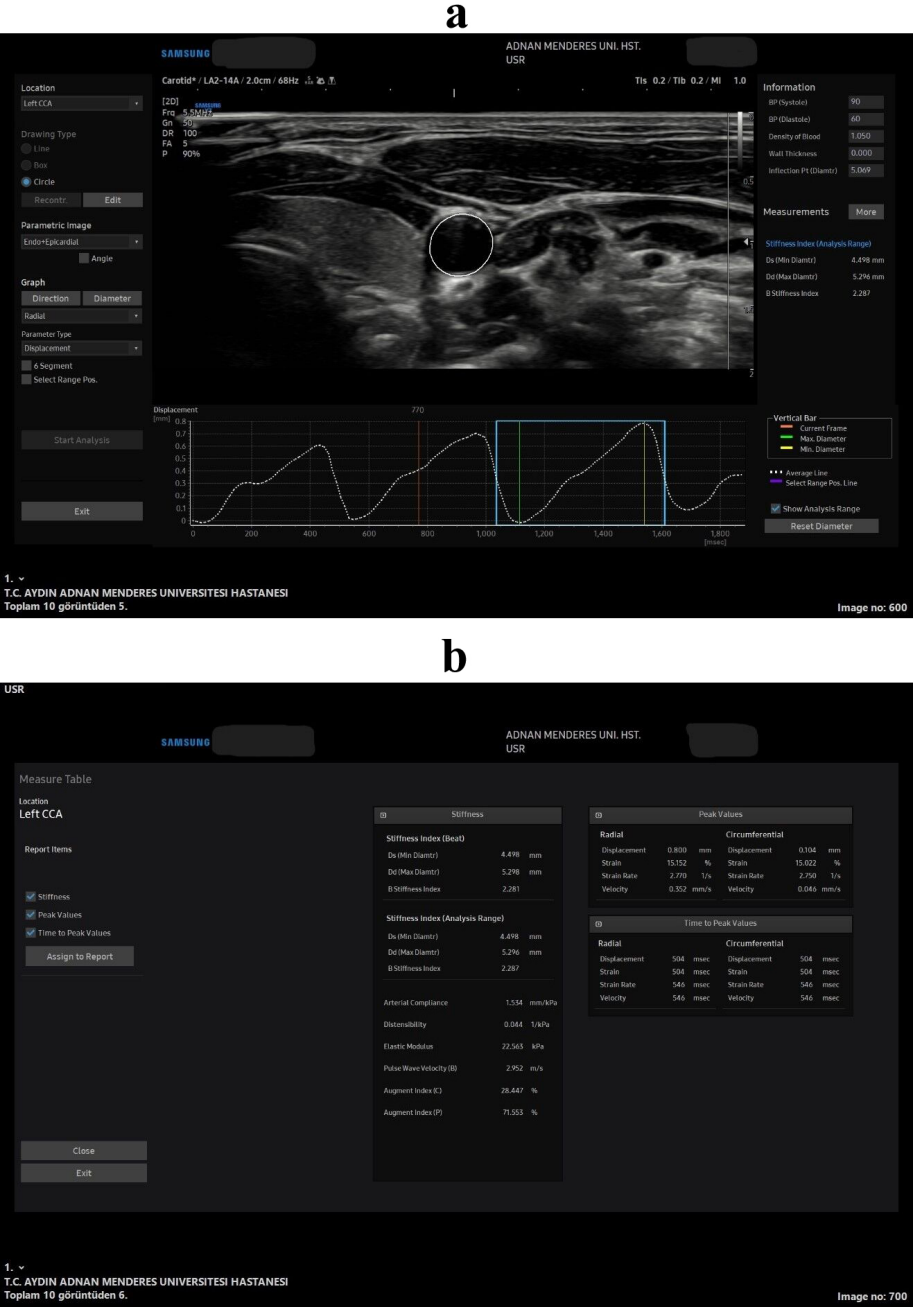
**All stiffness and strain measurements in the speckle tracking carotid strain (STCS) software in the axial plane**. Wave forms and the axial US image captured for the arterial analysis (a), all the measurements that was obtained from the soft-ware from that US wave form image (b). 2D, 2 Dimension; BP, blood pressure; Ds, diameter in systole; Dd, diameter in diastole; CCA, common carotis artery; US, ultrasonography.

**Fig. 2.**
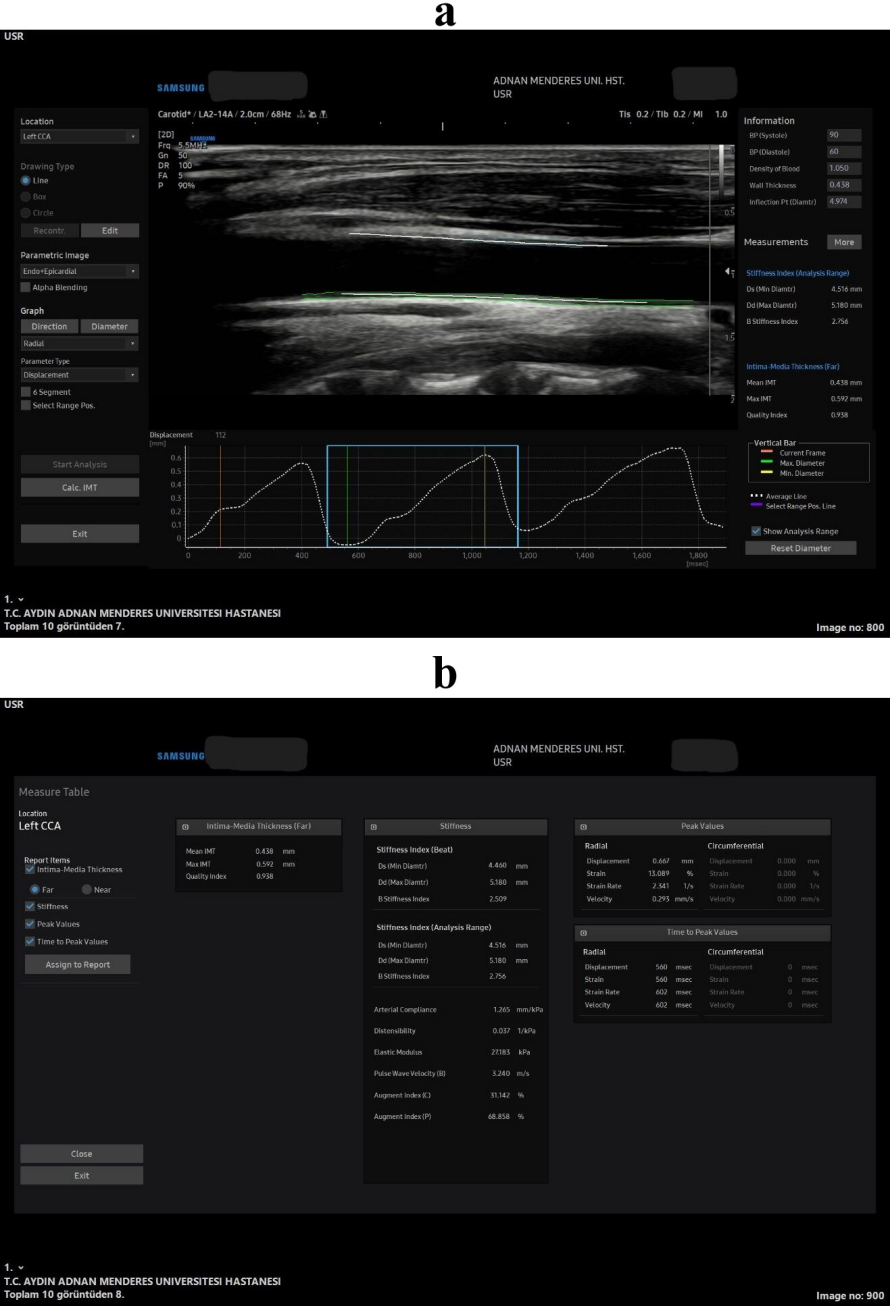
**All stiffness and strain measurements in the speckle tracking carotid strain (STCS) software in the longitudinal plane**. Wave forms and the longitudinal plane US image captured for the arterial analysis (a), all the measurements that was obtained from the soft-ware from that US wave form image (b).

Chronic inflammation in RA has been associated with endothelial dysfunction and arterial wall stiffness, which are considered critical early indicators of subclinical atherosclerosis [4]. Recent studies have demonstrated that inflammation associated with RA accelerates the development of atherosclerosis, thereby increasing the risk of CVD even in the absence of traditional risk factors [5, 6]. Patients with RA display elevated levels of inflammatory markers, including cytokines, which have been linked to the progression of atherosclerosis [7]. A specific subset of RA patients present increased CD4+ CD28- cell numbers, which promote immune activation and play a role in destabilizing atherosclerotic plaques, thereby precipitating acute cardiovascular events [8]. In recent years, there has been a significant increase in the utilization of non-invasive imaging modalities for assessing early cardiovascular changes in RA patients. Among these techniques, speckle tracking carotid strain (STCS) and ultrasonography (US) methods have gained particular attention due to the capacity of these techniques to evaluate arterial wall stiffness and strain in real time (Fig. 1) [9]. STCS enables the functional assessment of the carotid arteries, measuring changes in arterial wall stiffness and strain parameters, critical indicators of subclinical atherosclerosis. STCS, in conjunction with other parameters, such as intima-media thickness (IMT) (Fig. 2) and pulse wave velocity (PWV), offers valuable insights into cardiovascular risk in RA patients [10].

This study aimed to assess arterial wall stiffness and subclinical CVD in RA patients using non-invasive US and STCS software (RS80 Prestige V.3.01, L3-12A transducer, ArterAnalysis, Samsung, Medison Co., Ltd., Seoul, Korea). By evaluating parameters such as arterial wall strain, compliance, and stiffness indices, this research seeks to improve our understanding of the cardiovascular implications of RA and the role of advanced imaging techniques in detecting early atherosclerotic changes. The value of this approach lies in its non-invasive and cost-effective nature, which could enable earlier detection of cardiovascular risk and timely intervention, potentially reducing morbidity and mortality. Compared to traditional methods, such as IMT and PWV, STCS offers a more comprehensive assessment of arterial functions. Thus, this study aimed to enhance early detection strategies and contribute to more personalized cardiovascular risk management in RA patients, ultimately improving long-term outcomes in this high-risk population.

Table 1 summarizes the descriptions of all indicators, such as PWV, augmentation index (AI), arterial distensibility (AD), arterial compliance (AC), elastic modulus (EM), stiffness index (SI), and strain.

**Table 1.** **Arterial wall stiffness indicators**.

Parameters	Description
Arterial distensibility (AD)	Relative change in diameter concerning pressure increase
Arterial compliance (AC)	Absolute change in diameter in response to pressure increase
Elastic modulus (EM)	The pressure required for a 100% increase in basal diameter
Young elastic mod	Elastic modulus for each area
Pulse wave velocity (PWV)	Pulse propagation speed in the arterial system
Augmentation index (AI)	Increase in pressure after systolic peak
Beta stiffness index (β-SI)	Ratio of relative diameter changes from systolic to diastolic
Strain	Ratio of diameter changes under stress to basal diameter

## 2. Methods

### 2.1 Ethical Approval

This study was conducted in accordance with the ethical standards of the Institutional and National Research Committees and the 1964 Helsinki Declaration and its later amendments. The study protocol was approved by the Non-Interventional Clinical Research Ethics Committee of our Faculty of Medicine (approval date: 12.08.2021, decision number: 7).

### 2.2 Study Population and Sample Selection

This study included patients diagnosed with RA who presented to the Rheumatology Clinic of our Tertiary Care Hospital between July 2021 and July 2022. The sample size was calculated based on an a priori power analysis, which determined that at least 120 participants were required to achieve a statistical power of 80% with an effect size of 0.5 and a significance level (α) of 0.05. Consequently, 143 eligible patients who consented to participate were enrolled during the specified time frame, exceeding the minimum required sample size.

### 2.3 Inclusion and Exclusion Criteria

The inclusion criteria for this study comprised the following: patients diagnosed with RA in the Rheumatology Department of our institution, as well as healthy control participants matched by age and gender with no history of RA or other autoimmune or inflammatory diseases. Eligible participants were required to provide informed consent, be between 20 and 80 years old, not be pregnant, and have no history of carotid artery surgery or intervention. Individuals with no evidence of dense calcific plaques or complete carotid artery occlusion on imaging were also included.

The exclusion criteria were designed to complement the inclusion criteria and ensure the homogeneity of the study population. Participants were excluded if they had a history of carotid artery surgery or intervention, complete carotid artery occlusion, dense calcific plaques in the carotid artery, were pregnant, or were outside the age range of 20 to 80 years. These criteria aimed to minimize the impact of confounding variables on the study outcomes while ensuring accurate and reliable ultrasound measurements.

### 2.4 Data Collection

#### 2.4.1 Case Report Form

The researcher developed a case report form consisting of 11 questions based on a review of relevant literature and utilized as the primary data collection tool. The form collected comprehensive demographic and clinical information from the patients included in the study. Demographic data included age, gender, height, weight, and body mass index (BMI). Vital signs such as systolic blood pressure (SBP) and diastolic blood pressure (DBP) were also recorded, alongside smoking status and medication usage.

In addition, laboratory findings were obtained, including total cholesterol, low-density lipoprotein (LDL), high-density lipoprotein (HDL), triglycerides (TGs), hemoglobin (Hb), platelet count, erythrocyte sedimentation rate (ESR), C-reactive protein (CRP), disease activity score (DAS), anti-cyclic citrullinated peptide (anti-CCP), and rheumatoid factor (RF). The data were sourced from the hospital’s electronic record system, and patient follow-up files were archived in the hospital. All collected information was systematically documented in the case report form to ensure accurate and consistent data handling throughout the study.

#### 2.4.2 Calculation of Strain and Stiffness Parameters

A certified radiologist with expertise in vascular imaging conducted all carotid US examinations in this study. The operator underwent comprehensive training on using the US device and STCS software. Pilot tests were performed before the main research to ensure protocol standardization, and a predefined procedure was strictly followed during all US evaluations.

To enhance measurement consistency, a single operator performed all analyses. The measurement protocols were based on established standards in the literature. For instance, a specific segment of the common carotid artery was selected for analysis, and measurements were conducted in axial and longitudinal planes as part of the protocol.

Carotid US examinations were performed using the Samsung RS80 US device with an L3-12A linear probe (Samsung Medison Co., Ltd., Seoul, Korea). The arterial analysis software (Samsung Medison Co., Ltd., Seoul, Korea) was employed to calculate strain and stiffness parameters. All the parameters estimated by the software are described in Table 1. Displacement of the common carotid artery (CCA) was automatically calculated to assess its functional capacity, with the segment just below the carotid bulb selected for analysis. The operator manually determined the control points on the CCA, and the arterial wall displacement was tracked using an optical flow algorithm integrated into the software.

Before the US examination, patients rested in a supine position for 10 minutes. Afterward, systolic and diastolic blood pressure and pulse rate were measured using a Reister sphygmomanometer (Reister 1312 Minimus II, Rudolf Riester GmbH, Jungingen, Germany). These values and the patient’s height and weight were entered into the arterial analysis software. The CCA was evaluated in both axial (Fig. 1) and longitudinal planes (Fig. 2), with the mean values of each measurement recorded for both the right and left CCA. All images and measurements were obtained during the research session—after the 10-minute rest period—ensuring that the image display time falls within the defined research time range.

The software automatically measured all strain and stiffness parameters in the axial (Fig. 1) and longitudinal (Fig. 2) planes. The carotid intima-media thickness (CIMT) was also assessed using the same software with the quality index (QI) measurement in the longitudinal plane only (Fig. 2). To achieve this, the interfaces of the blood–intima boundaries within the carotid artery (at least five points in total) were identified on a static image for both the anterior and posterior walls. The software automatically tracked the movement of these points to calculate the relevant parameters.

### 2.5 Statistical Analysis

The research data were analyzed using SPSS version 21.0 (IBM Corp., Armonk, NY, USA). The normality of the continuous variables was assessed using the Shapiro–Wilk test and visual inspections (e.g., histograms and probability plots). For variables with a normal distribution, data are presented as the mean ± standard deviation, and comparisons were performed using the student’s *t*-test. For variables that did not follow a normal distribution, data are presented as the median (minimum–maximum), and comparisons were conducted using the Mann–Whitney U test. To ensure clarity and reproducibility, the choice of statistical tests was explicitly based on the normality assessment. Two-tailed *p*-values are reported unless otherwise specified.

The Chi-square test was employed for categorical variables to evaluate differences between groups. Continuous variables with parametric distributions were compared between independent groups using the student’s *t*-test, while the Mann–Whitney U test was utilized for non-parametric continuous variables. This study used Pearson’s correlation test for parametric variables that met the assumptions of normality and linearity, while Spearman’s correlation test was employed for variables that did not meet these assumptions or where monotonic relationships were of interest. Although Spearman’s test is commonly associated with non-parametric variables, it is not limited to these and can also be used for ranked data or monotonic relationships in parametric datasets. This approach ensured that the most appropriate statistical method was applied based on the characteristics of the data, enhancing the robustness of the analysis.

The multivariable linear regression model in this study was designed to evaluate the relationship between RA and arterial stiffness and strain parameters, with RA as the independent variable (X) and stiffness and strain parameters as the dependent continuous variables (Y). The model was adjusted for potential confounders, including age, gender, BMI, and smoking status. These were selected based on their well-established roles as cardiovascular risk factors in the literature, independent of their correlation with the primary variables. Additionally, variables showing significant associations in univariate analyses were included to assess their contribution alongside the identified confounders. This comprehensive approach ensures that the independent effect of RA on vascular stiffness and strain is accurately evaluated, accounting for potential confounding influences. Statistical tests were conducted using two-tailed *p*-values to detect differences in either direction, with a significance threshold of *p* < 0.05. Specific regression results, including β-coefficients, confidence intervals, and *p*-values, are detailed in Table 2 to provide clarity and transparency regarding the findings.

**Table 2.** Multivariate linear regression analysis*.

		β	95% Confidence interval for β		*p*-value
			Lower bound	Upper bound	
CIMT, mean		0.050	0.017	0.082	**0.003**
Axial plane					
Stiffness parameters					
	β-SI	–2.460	–3.619	–1.301	< **0.001**
	AC (mm/kPa)	0.098	0.016	0.179	**0.019**
	AD (/kPa)	0.002	0.001	0.003	**0.001**
	EM (kPa)	–39.708	–56.739	–22.676	< **0.001**
	PWV (m/s)	–0.711	–1.141	–0.280	**0.001**
Strain parameters (radial)					
	DP (mm)	0.048	0.013	0.082	**0.007**
	Strain (%)	0.834	0.355	1.313	**0.001**
	SR (1/s)	0.105	0.049	0.162	< **0.001**
Strain parameters (circumferential)					
	DP (mm)	0.003	–0.002	0.009	0.226
	Strain (%)	0.819	0.342	1.295	**0.001**
	SR (1/s)	0.103	0.047	0.159	< **0.001**
Longitudinal plane					
Stiffness parameters					
	β-SI	–2.598	–3.775	–1.421	< **0.001**
	AC (mm/kPa)	0.144	0.069	0.218	< **0.001**
	AD (/kPa)	0.003	0.001	0.004	< **0.001**
	EM (kPa)	–41.039	–59.048	–23.029	< **0.001**
	PWV (m/s)	–0.862	–1.304	–0.419	< **0.001**
Strain parameters (radial)					
	DP (mm)	0.064	0.028	0.100	**0.001**
	Strain (%)	1.118	0.557	1.679	< **0.001**
	SR (1/s)	0.102	0.033	0.171	**0.004**

*Adjusted for age, gender, body mass index, and presence of smoking. CIMT, carotid intima-media thickness; DP, displacement; SR, strain rate. Bold *p*-values represent *p* < 0.05.

## 3. Results

A total of 143 participants were included in the study, of which 75 were RA patients (60 females and 15 males) and 68 were healthy controls (54 females and 14 males). The mean age of the RA group was 50.9 ± 11.4 years (range: 25.0–74.0), and their mean BMI was 27.6 ± 4.6 kg/m^2^. In contrast, the control group had a mean age of 53.1 ± 12.6 years (range: 20.0–77.0) and a mean BMI of 26.3 ± 4.9 kg/m^2^. Table 3 provides a detailed comparison of the demographic characteristics and vital signs of the RA and control groups, showing a statistically significant difference in SBP, which was higher in the RA group (126.5 ± 17.8 mmHg) compared to the control group (118.2 ± 12.7 mmHg; *p* < 0.05). DBP was similar between the groups, with mean values of 78.3 ± 9.7 mmHg for the RA group and 75.6 ± 9.1 mmHg for the controls.

**Table 3.** **Comparison of the sociodemographic characteristics of cases in the RA and control groups**.

	Group				*p*-value
	RA (n = 75)		Control (n = 68)		
	Mean ± SD	Median (min–max)	Mean ± SD	Median (min–max)	
Age^a^ (year)	50.9 ± 11.4	50.0 (25.0–74.0)	53.1 ± 12.6	53.0 (20.0–77.0)	0.262^a^
Height^a^ (cm)	164.8 ± 6.6	165.0 (151.0–181.0)	164.3 ± 9.1	163.5 (150.0–193.0)	0.309^a^
Weight^b^ (kg)	74.7 ± 12.6	74.0 (47.0–102.0)	70.8 ± 12.8	70.0 (45.0–117.0)	0.066^b^
BMI^b^ (kg/m^2^)	27.6 ± 4.6	27.5 (17.0–38.9)	26.3 ± 4.9	26.1 (17.5–39.1)	0.127^b^
SBP^a^ (mmHg)	126.5 ± 17.8	126.0 (100.0–230.0)	118.2 ± 12.7	120.0 (90.0–150.0)	**0.003^a^**
DBP^b^ (mmHg)	78.3 ± 9.7	80.0 (60.0–110.0)	75.6 ± 9.1	80.0 (60.0–100.0)	0.244^b^

RA, rheumatoid arthritis; BMI, body mass index; SBP, systolic blood pressure; DBP, diastolic blood pressure; SD, standard deviation; ^a^, independent samples *t*-test used for normally distributed data (presented as the mean ± SD); ^b^, Mann–Whitney U test used for non-normally distributed data (presented as the median (min–max)). Normality was assessed using the Shapiro–Wilk test. Bold *p*-values represent *p* < 0.05.

Laboratory findings of both groups revealed that CRP levels were significantly higher in the RA group compared to the control group (*p* < 0.05). No statistically significant differences were found between the groups for other laboratory parameters such as total cholesterol, LDL, HDL, TGs, hemoglobin (Hb), and platelet count (all *p* > 0.05) (Table 4). Table 5 highlights key laboratory parameters and disease-specific features in the RA group, which are critical for understanding the relationship between RA disease activity and cardiovascular risk markers assessed in the study.

**Table 4.** **Comparison of laboratory parameters between cases in the RA and control groups**.

	Group						*p*-value
	RA			Control			
	n	Mean ± SD	Median (min–max)	n	Mean ± SD	Median (min–max)	
Total cholesterol^a^	73	203.3 ± 39.9	200.0 (120.0–321.0)	51	198.3 ± 42.0	201.0 (94.0–276.0)	0.501^a^
LDL^a^	73	114.2 ± 32.9	105.0 (49.0–223.0)	51	116.7 ± 35.5	115.0 (39.0–190.0)	0.536^a^
HDL^a^	73	66.8 ± 16.2	64.3 (35.0–105.0)	51	61.2 ± 18.0	60.2 (27.6–94.1)	0.071^a^
TG^b^	73	109.6 ± 45.4	95.0 (33.0–272.0)	51	115.2 ± 49.9	109.0 (48.0–253.0)	0.549^b^
Hb^a^	75	12.9 ± 1.3	12.7 (10.4–16.8)	64	16.4 ± 19.4	13.1 (8.5–123.0)	0.263^a^
Platelet^a^	75	291,893.3 ± 76,580.2	296,000.0 (113,000.0–458,000.0)	64	284,562.5 ± 88,033.0	273,000.0 (96,000.0–553,000.0)	0.600^a^
ESR^b^	75	31.8 ± 16.9	31.0 (4.0–68.0)	47	30.1 ± 25.5	22.0 (2.0–137.0)	0.101^b^
CRP^b^	72	7.4 ± 11.5	3.3 (2.0–71.9)	53	8.6 ± 22.2	2.0 (0.8–145.0)	**0.033^b^**

min, minimum; max, maximum; LDL, low-density lipoprotein; HDL, high-density lipoprotein; TG, triglyceride; Hb, hemoglobin; ESR, erythrocyte sedimentation rate; CRP, C-reactive protein. Normality was assessed by the Shapiro–Wilk test. Bold *p*-values represent *p* < 0.05.

**Table 5.** **Laboratory and clinical characteristics of the RA cohort**.

DAS (mean ± SD)			3.13 ± 0.81
	Anti-CCP, n (%)	Negative	13 (19.7)
		Positive	53 (80.3)
Anti-CCP (mean ± SD)			78.42 ± 81.76
	RF, n (%)	Negative	16 (24.6)
		Positive	49 (75.4)
RF (mean ± SD)			136.30 ± 200.29
	Smoking, n (%)	No	58 (77.3)
		Yes	17 (22.7)
Duration after diagnosis (months) (mean ± SD)			82.99 ± 95.01

DAS, disease activity score; Anti-CCP, antibodies cyclic citrullinated peptide; RF, rheumatoid factor; n, number.

Table 5 presents laboratory parameters specific to the RA group, including DAS, anti-CCP, RF, smoking status, and duration after diagnosis. These variables provide insights into the clinical characteristics of the RA cohort, supporting a deeper understanding of disease activity and its relationship with cardiovascular risk markers.

Meanwhile, Tables 6,7 compare the RA and control groups regarding arterial wall stiffness and strain. For variables with a normal distribution, the mean ± SD is reported, and an independent samples *t*-test was used; for non-normally distributed data, the median (min–max) and the Mann–Whitney U test are presented.

**Table 6.** **Comparison of stiffness and strain parameters in the longitudinal plane between the RA and control groups**.

		Group								*p*-value
		RA (n = 75)				Control (n = 68)				
		Mean ± SD	Median	Min	Max	Mean ± SD	Median	Min	Max	
CIMT mean^a^		0.541 ± 0.121	0.535	0.346	1.068	0.604 ± 0.159	0.592	0.305	1.096	**0.006^a^**
CIMT QI^b^		0.884 ± 0.139	0.958	0.423	1.000	0.872 ± 0.162	0.949	0.381	1.000	0.493^b^
Stiffness parameters										
	β-SI^a^	9.937 ± 4.655	8.330	3.363	23.548	7.298 ± 1.950	7.041	3.283	12.686	**0.001^a^**
	AC (mm/kPa)^b^	0.519 ± 0.209	0.512	0.135	1.093	0.665 ± 0.231	0.655	0.307	1.326	**0.000^b^**
	AD (/kPa)^b^	0.009 ± 0.004	0.010	0.002	0.025	0.012 ± 0.004	0.011	0.006	0.032	**0.000^b^**
	EM (kPa)^a^	134.357 ± 73.181	111.180	42.235	461.259	93.008 ± 27.494	90.944	32.380	165.550	**0.000^a^**
	PWV (m/s)^b^	6.676 ± 1.758	6.351	0.000	12.113	5.822 ± 0.828	5.875	3.520	7.906	**0.001^b^**
Strain parameters (radial)										
	DP (mm)^b^	0.343 ± 0.101	0.327	0.155	0.616	0.406 ± 0.110	0.400	0.254	0.859	**0.001^b^**
	Strain (%)^b^	5.678 ± 1.719	5.451	2.578	11.051	6.757 ± 1.645	6.484	4.140	12.751	**0.000^b^**
	SR (1/s)^b^	0.592 ± 0.209	0.561	0.223	1.169	0.698 ± 0.217	0.693	0.309	1.698	**0.001^b^**

QI, quality index. Note: Normality was assessed using the Shapiro–Wilk test. The choice of mean ± SD vs. median (min–max) and the corresponding statistical tests were based on the normality distribution of each variable. Bold *p*-values represent *p* < 0.05.

**Table 7.** **Comparison of stiffness and strain parameters in the axial plane between the RA and control groups**.

		Group								*p*-value
		RA (n = 75)				Control (n = 68)				
		Mean ± SD	Median	Min	Max	Mean ± SD	Median	Min	Max	
Stiffness parameters										
	β-SI^a^	10.319 ± 4.726	9.092	4.023	24.693	8.001 ± 2.513	7.233	3.585	14.771	**0.004^a^**
	AC (mm/kPa)^b^	0.675 ± 0.258	0.637	0.215	1.654	0.759 ± 0.257	0.736	0.323	1.539	**0.036^b^**
	AD (/kPa)^b^	0.009 ± 0.004	0.008	0.002	0.020	0.011 ± 0.004	0.011	0.006	0.029	**0.001^b^**
	EM (kPa)^a^	139.505 ± 71.890	120.937	50.528	473.135	101.938 ± 34.061	95.897	35.365	182.030	**0.001^a^**
	PWV (m/s)^b^	6.789 ± 1.737	6.741	0.000	12.267	6.098 ± 1.001	6.001	3.686	8.192	**0.003^b^**
Strain parameters (radial)										
	DP (mm)^b^	0.376 ± 0.103	0.368	0.056	0.797	0.416 ± 0.109	0.403	0.191	0.697	**0.028^b^**
	Strain (%)^b^	5.474 ± 1.502	5.170	2.930	11.272	6.201 ± 1.671	6.053	3.268	11.807	**0.006^b^**
	SR (1/s)^b^	0.594 ± 0.185	0.573	0.340	1.346	0.688 ± 0.219	0.665	0.389	1.610	**0.004^b^**
Strain parameters (circumferential)										
	DP (mm)^b^	0.051 ± 0.019	0.048	0.032	0.177	0.054 ± 0.014	0.053	0.025	0.091	0.074^b^
	Strain (%)^b^	5.451 ± 1.487	5.102	2.873	11.151	6.166 ± 1.675	5.981	3.168	11.724	**0.007^b^**
	SR (1/s)^b^	0.592 ± 0.184	0.560	0.343	1.366	0.684 ± 0.217	0.667	0.391	1.589	**0.004^b^**

Normality was assessed using the Shapiro–Wilk test. Mean ± SD was used for descriptive purposes if data were normally distributed, whereas median (min–max) was reported for non-normally distributed data. Group comparisons were performed with the corresponding test indicated above. Bold *p*-values represent *p* < 0.05.

Stiffness parameters: The RA group consistently exhibited higher β-SI, EM, and PWV values than the control group (all *p* < 0.05), indicating increased arterial stiffness in RA patients. In contrast, compliance and distensibility (AC and AD) were lower in the RA group (*p* < 0.05), reflecting decreased vessel elasticity.

Strain parameters: Most radial and circumferential strain indices (displacement (DP), strain %, strain rate (SR)) were significantly lower in RA patients (*p* < 0.05), suggesting reduced arterial wall deformation capacity compared to controls. However, the axial plane circumferential DP parameter did not differ significantly between RA patients and controls (*p* = 0.074).

These findings underscore a pattern of increased stiffness and reduced compliance/strain in patients with RA. Only the axial plane circumferential DP did not reach statistical significance.

Tables 8,9 examine the correlations of stiffness and strain parameters with the DAS and disease duration in RA patients. Table 8 focuses on parameters in the longitudinal plane, while Table 9 presents those in the axial plane. The DAS reflects RA disease activity, and disease duration refers to the time elapsed since RA diagnosis. Among our 75 patients, the shortest recorded duration was 1 month, and the longest was 480 months, with a mean of 81.88 months. No significant correlation was found between the DAS and any stiffness or strain parameters in both planes. Regarding disease duration, only the CIMT mean parameter in the longitudinal plane (Table 8) showed a statistically significant correlation (r = 0.373, *p* = 0.001). In contrast, none of the axial plane parameters (Table 9) were significantly correlated with disease duration. The heterogeneity of the RA population included in the study may influence these results.

**Table 8.** **Correlation between stiffness and strain parameters in the longitudinal plane with DAS and disease duration in RA patients**.

			DAS	Disease duration after diagnosis
CIMT mean		r	–0.156	0.373
		*p*	0.181	**0.001**
Stiffness parameters				
	β-SI	r	–0.099	–0.019
		*p*	0.396	0.873
	AC (mm/kPa)	r	0.051	0.086
		*p*	0.667	0.469
	AD (/kPa)	r	0.094	–0.009
		*p*	0.423	0.939
	EM (kPa)	r	–0.095	0.020
		*p*	0.419	0.867
	PWV (m/s)	r	–0.198	0.102
		*p*	0.089	0.387
Strain parameters (radial)				
	DP (mm)	r	0.071	0.055
		*p*	0.547	0.642
	Strain (%)	r	0.121	–0.013
		*p*	0.303	0.910
	SR (1/s)	r	0.091	–0.081
		*p*	0.439	0.493

Bold *p*-values represent *p* < 0.05. Disease duration refers to the time elapsed since RA diagnosis.

**Table 9.** **Correlation between stiffness and strain parameters in the axial plane with DAS and duration after RA diagnosis**.

			DAS	Disease duration after diagnosis
Stiffness parameters				
	β-SI	r	–0.112	0.070
		*p*	0.337	0.555
	AC (mm/kPa)	r	0.035	–0.037
		*p*	0.767	0.755
	AD (/kPa)	r	0.162	–0.131
		*p*	0.166	0.264
	EM (kPa)	r	–0.139	0.104
		*p*	0.234	0.377
	PWV (m/s)	r	–0.209	0.120
		*p*	0.071	0.307
Strain parameters (radial)				
	DP (mm)	r	0.060	–0.064
		*p*	0.609	0.586
	Strain (%)	r	0.147	–0.131
		*p*	0.208	0.265
	SR (1/s)	r	0.127	–0.186
		*p*	0.279	0.112
Strain parameters (circumferential)				
	DP (mm)	r	0.094	–0.073
		*p*	0.424	0.535
	Strain (%)	r	0.148	–0.130
		*p*	0.204	0.270
	SR (1/s)	r	0.137	–0.182
		*p*	0.240	0.120

The disease duration reflects the time elapsed since the clinical diagnosis of RA.

In the multivariate linear regression analysis adjusted for age, BMI, and smoking, RA was negatively and independently associated with axial and longitudinal β-SI, EM, and PWV (Table 2). In the same analysis, RA was positively and independently associated with both axial and longitudinal AC, AD, and all strain parameters, except for the circumferential DP parameter in the axial plane (*p* = 0.226), which was not statistically significant (all other *p* < 0.05). The 95% confidence intervals for the β-coefficients indicate the range within which the true association is expected to lie with a 95% confidence level.

## 4. Discussion

This study aimed to investigate the influence of RA on arterial stiffness and strain parameters using a novel, non-invasive imaging technique, STCS–US. Our findings demonstrated that RA patients exhibit significantly higher arterial stiffness and altered strain parameters compared to healthy controls, emphasizing the role of chronic inflammation in vascular remodeling. Using STCS–US, a novel imaging modality, we detected functional changes in the carotid arteries that precede structural changes such as plaque formation. These results align with previous studies assessing vascular stiffness in RA patients and highlight the potential of STCS–US in early cardiovascular risk stratification. Unlike traditional markers, stiffness and strain parameters offer a more sensitive evaluation of vascular health, providing a pathway for earlier intervention.

RA is a systemic, chronic inflammatory disease that primarily affects the joints. RA is associated with an increased risk of cardiovascular events. The mortality, adjusted for age and sex, is 0.5–2 times higher in RA patients than in healthy individuals, and CVD is responsible for about 50–60% of this mortality [11, 12]. Subsequently, the effects of RA on the cardiovascular system have become a growing area of interest [13]. Chronic inflammation, a hallmark of RA, promotes endothelial dysfunction, augmented arterial wall stiffness, and subclinical atherosclerosis, all early markers of cardiovascular events [14]. This study aimed to investigate the influence of RA on arterial wall stiffness and strain parameters using a novel, non-invasive imaging technique, STCS–US. By assessing these vascular parameters, we intended to identify early cardiovascular changes in patients with RA and compare them with a demographically matched control group.

The current literature on arterial wall stiffness and RA contains many original articles, systematic reviews, and meta-analyses [15, 16]. The most common technique in those studies was applanation tonometry; very few studies used STCS–US technology in the RA group [9]. While our research builds upon the foundational work of Lee *et al*. (2012) [9], who first applied speckle tracking strain imaging to assess carotid arterial stiffness in RA patients, our study extends this approach in several significant ways. Firstly, we conducted a comprehensive parameter analysis by examining a broader range of stiffness and strain metrics, including β-SI, AC, AD, EM, and PWV. This extensive analysis provides a more thorough assessment of arterial health than previous studies. Secondly, we employed the latest STCS–US technology, which offers enhanced resolution and accuracy over earlier systems, thereby enabling more precise measurements of arterial properties. Additionally, our research incorporates multivariate linear regression analysis adjusted for potential confounders such as age, gender, BMI, and smoking status, allowing for a more robust evaluation of the independent effects of RA on arterial stiffness. Unlike Lee *et al*. (2012) [9], this study assessed arterial parameters in both longitudinal and axial planes, offering a more comprehensive view of arterial dynamics. Furthermore, we explored the correlation between arterial stiffness parameters and RA disease activity score measures, including DAS28 and disease duration, providing valuable insights into the progression of vascular changes in RA patients. These distinctions enhance our understanding of arterial stiffness in the context of RA and contribute to the evolving body of knowledge in vascular health research. Applanation tonometry techniques are time-consuming, require dedicated equipment, and are not widely used in clinical practice [17, 18]. In contrast to applanation tonometry, measuring local stiffness using STCS provides additional information about arterial wall compliance and local changes in the heterogeneous movement pattern; thus, STCS assures a superior index of whole artery wall stress [19, 20].

### 4.1 Chronic Inflammation and Cardiovascular Risk in RA

Chronic inflammation is well-established as a critical driver of atherosclerosis and other cardiovascular complications in RA patients [21]. Several studies have shown that inflammatory markers, particularly CRP, are elevated in RA patients and are directly associated with increased cardiovascular risk [22]. In our study, CRP levels were significantly higher in the RA group compared to the control group, which is consistent with the current literature. This finding also aligns with the work of Myasoedova *et al*. [23], who demonstrated that serum CRP levels in RA patients frequently exceed the 3 mg/L and 10 mg/L cutoffs associated with high and very high cardiovascular risk in the general population. CRP reflects the inflammatory burden in RA and acts as an independent predictor of cardiovascular events [24]. Interestingly, our study found minimal differences in other lipid markers, such as total cholesterol, LDL, HDL, and TGs, between RA patients and controls, which is consistent with the findings of Erum *et al*. [25]. This phenomenon in RA, known as the “lipid paradox”, suggests that the relationship between lipids and cardiovascular risk in RA patients is more complex than in the general population. The role of inflammation in endothelial dysfunction is key, as it leads to arterial wall stiffening, which precedes the development of structural atherosclerotic changes, such as carotid plaque formation. This is supported by Popescu *et al*. [26], who found that RA patients have increased arterial wall stiffness compared to healthy controls, as measured by PWV.

### 4.2 Blood Pressure and RA

Hypertension is a well-documented cardiovascular risk factor in RA patients, likely stemming from the same inflammatory pathways that drive atherosclerosis. Several studies have reported elevated blood pressure in RA patients, potentially linked to vascular endothelial dysfunction [27]. Bedeković *et al*. [28] found that the prevalence of hypertension in RA patients was significantly higher than in the general population, with rates ranging from 52% to 73%.

In a study by Manavathongchai *et al*. [27], significantly higher SBP was observed in RA patients compared to controls (129 ± 17 vs. 124 ± 16 mmHg, respectively; *p* = 0.002). This finding aligns with other research demonstrating altered vascular function in RA. Endothelial dysfunction, driven by chronic inflammation, leads to impaired nitric oxide (NO) production and vascular smooth muscle cell reactivity, contributing to the development of hypertension [29]. Hansildaar *et al*. [30] demonstrated that RA patients exhibit significantly lower flow-mediated dilation, indicating endothelial dysfunction.

The increase in blood pressure is clinically significant as it exacerbates the already heightened cardiovascular risk in RA patients. Indeed, a paper by Jagpal and Navarro-Millán [31] showed that hypertension in RA was associated with an 84% increased risk of cardiovascular events (relative risk (RR) 1.84, 95% CI 1.38–2.46), thereby underscoring the importance of monitoring and managing blood pressure in RA patients to reduce their long-term cardiovascular morbidity and mortality.

The European League Against Rheumatism (EULAR) recommendations for cardiovascular disease risk management emphasize the need for regular screening and management of modifiable cardiovascular risk factors in RA patients, including hypertension [32]. These guidelines suggest considering a blood pressure target <130/80 mmHg in RA patients, highlighting the importance of tight blood pressure control in this high-risk population [32].

In conclusion, hypertension is a significant comorbidity in RA patients that requires careful attention and management to mitigate the increased cardiovascular risk associated with the disease. Moreover, regular monitoring and appropriate treatment of hypertension should be an integral part of the comprehensive care of RA patients. In our study, even though our RA patients were well monitored for hypertension, RA patients still had statically higher SBP than the control group (Table 3; *p* < 0.05).

### 4.3 Arterial Wall Stiffness and Vascular Health in RA

Arterial wall stiffness is a key marker of early vascular aging and is known to precede the development of atherosclerotic plaques. Furthermore, increased arterial wall stiffness is associated with a higher risk of cardiovascular events, including myocardial infarction and stroke [33]. Our study used STCS–US to assess arterial wall stiffness and strain parameters in RA patients and healthy controls. We found significant differences in all the stiffness and strain parameters between the two groups, with RA patients showing a statistically significant increase in arterial wall stiffness (*p* < 0.05). This is consistent with findings from previous studies, which reported increased carotid IMT and arterial wall stiffness in RA patients [9, 34]. The increase in arterial wall stiffness may directly result from chronic inflammation, which leads to collagen deposition and vascular wall remodeling, reducing its elasticity [34].

Carotid IMT is a well-established marker of subclinical atherosclerosis, and its increase is often observed in patients with RA. Our study demonstrated that the control group had significantly higher mean carotid IMT values compared to RA patients (mean ± SD: controls = 0.604 ± 0.159 mm vs. RA = 0.541 ± 0.121 mm; *p* = 0.006), which is consistent with the current literature [35]. IMT is correlated with the risk of coronary artery disease (CAD) and cerebrovascular events, making it a critical marker for cardiovascular risk [34]. However, while IMT measures arterial structural changes, stiffness parameters assessed by STCS provide functional insights that often precede these structural changes. For example, a study by van Breukelen *et al*. [36] demonstrated that strain parameters may be more sensitive than IMT in detecting early vascular changes, particularly in populations at risk for atherosclerosis.

### 4.4 Clinical Implications and Future Directions

The findings of this study have important clinical implications. RA patients are at a significantly increased risk of CVDs, and early detection of subclinical vascular changes is critical in preventing long-term cardiovascular events. Non-invasive imaging techniques, such as STCS, provide a valuable tool for early cardiovascular risk stratification. Since arterial wall stiffness and strain parameters often change before structural markers, such as IMT, are detected, incorporating these assessments into routine clinical practice could improve the early detection of cardiovascular risk in RA patients.

Furthermore, cardiovascular risk management in RA should be a multidisciplinary effort involving rheumatologists, cardiologists, and primary care providers. Aggressive inflammation management using disease-modifying antirheumatic drugs (DMARDs) and biologics, alongside traditional cardiovascular risk factors, such as hypertension and dyslipidemia, is essential. Longitudinal studies are needed to determine whether improvements in arterial wall stiffness and strain parameters correlate with reduced cardiovascular events over time, particularly in RA patients receiving biologic therapies that target inflammatory pathways.

### 4.5 Limitations

This study has several limitations. First, its cross-sectional design prevents us from establishing a causal relationship between RA and increased arterial wall stiffness and strain. Thus, longitudinal studies are needed to evaluate whether the early vascular changes observed in this study predict future cardiovascular events. Even though this study adjusted for confounding factors such as age, gender, BMI, and smoking by implementing a multivariate linear regression analysis, other variables, such as treatment regimens and comorbidities, including diabetes or hypertension, were not fully accounted for. These factors could influence the observed vascular outcomes and should be considered in future studies. Additionally, a single radiologist performed our measurements only once; thus, no intra- and inter-observer variability evaluation could be conducted. Finally, the sample size, while sufficient for detecting significant differences, could be expanded in future research to improve the generalizability of the findings.

## 5. Conclusion

This study highlights the significant impact of RA on arterial wall stiffness and strain, markers of early cardiovascular risk. STCS–US can provide valuable insights into both functional and structural changes in the carotid arteries of RA patients, demonstrating that RA accelerates vascular aging and increases cardiovascular risk. These findings underscore the importance of early cardiovascular assessment and intervention in RA patients to mitigate the long-term burden of cardiovascular disease.

## Availability of Data and Materials

All the data generated during the study is presented in the results section.
